# Isolation and Identification of Methicillin-Resistant *Staphylococcus aureus* (MRSA) from Milk in Shire Dairy Farms, Tigray, Ethiopia

**DOI:** 10.1155/2020/8833973

**Published:** 2020-08-15

**Authors:** Weldemelak Girmay, Getachew Gugsa, Habtamu Taddele, Yisehak Tsegaye, Nesibu Awol, Meselu Ahmed, Aklilu Feleke

**Affiliations:** ^1^College of Veterinary Sciences, Mekelle University, Mekelle, Ethiopia; ^2^School of Veterinary Medicine, Wollo University, Dessie, Ethiopia; ^3^Aklilu Lemma Institute of Pathobiology, Addis Ababa University, Addis Ababa, Ethiopia

## Abstract

Antibiotic-resistant *Staphylococcus aureus* isolates pose a severe challenge to both veterinary and health professions and dairy cattle producers. Cross-sectional study was conducted from November 2014 to May 2015 to isolate and identify *S. aureus* from mastitic cows' milk and estimate the occurrence of MRSA in the dairy farms of Shire. Physical examination and California mastitis test were performed on a total of 220 dairy cows. Bacteriological isolation and identification and antibiogram testing were performed. Furthermore, multiplex polymerase chain reaction (PCR) was done for the detection of *mec A* and *fem A* genes. Out of the 220 dairy cows, 64 (29.09%) were positive for bovine mastitis, and of these, 32.81% were coagulase-positive *S. aureus* (CoPS). Antibiogram test results showed 100% of the isolates were resistant to penicillin G, nalidixic acid, and ampicillin, and 33.33% of the CoPS showed resistance to oxacillin (phenotypically MRSA positive). But 38.09% of the CoPS were found to be resistant and susceptible to vancomycin. PCR amplification of the seven phenotypically MRSA isolates revealed that 42.9% and 71.4% of them were found to carry *fem A* and *mec A* genes, respectively. The current study revealed the existence of alarming level of CoPS and development of multidrug resistance.

## 1. Introduction

Roughly 75% of the infectious diseases in humans over the past two decades have originated from animals and can transmit directly or indirectly from animals to humans [1]. *Staphylococcus aureus* (*S. aureus*) is recognized worldwide as a leading pathogen causing many serious diseases in dairy and healthcare surroundings [2]. In the last few decades, Staphylococcal food poisoning has been reported as the third cause of food-borne illnesses in the world [3]. Moreover, antibiotic-resistant *S. aureus* isolates pose a severe challenge to both veterinary and health professions and dairy cattle producers because they have a negative impact on therapy [4]. In the 1960s, approximately 80% of all clinical isolates of *S. aureus* were *β*-lactamase producers [5]. In the early 1960s, the first isolates of MRSA were detected in the United Kingdom (UK) [6].

The antibiotic resistance mechanism of MRSA is mediated through expression of *mec A*, encoding a penicillin-binding protein PBP2a that has a low affinity for *β*-lactam antibiotics [7]. The *mec A* gene is a DNA segment of 2.1 kb that is non-native to *S. aureus* and is inserted in a large block of exogenous DNA, known as the staphylococcal cassette chromosome *mec* (SCC*mec*) [8].

Infections due to MRSA have increased worldwide during the past twenty years [9]. Traditionally, two groups are distinguished among its strains: hospital-acquired MRSA (HA-MRSA) and community-acquired MRSA (CA-MRSA) [10]. However, MRSA infection and colonization have also been reported in horses, dogs, cats, birds, dairy cows, and chicken, and this new type of isolate emerged into the *S. aureus* population, belonging to clonal complex 398 (CC398) and is known as livestock-associated MRSA (LA-MRSA) [11, 12]. Hence, besides its importance as a hospital and community pathogen, MRSA has also been considered as an emerging problem in veterinary medicine in recent years [13, 14].

In recent years, MRSA strains have been recovered from several animal-source foods, such as poultry, pork, and beef, suggesting that foods may serve as the reservoir and source of CA-MRSA [15]. Milk has been reported to be contaminated with MRSA, which is suspected to have been acquired from dairy animals or from postmilking contamination and poor sanitary practice [16]. In addition, *S. aureus* is considered as one of the most important causative agents of mastitis in dairy cattle [17]. Hence, determination of antimicrobial resistance profile of LA-MRSA isolates is crucial for empirical treatment of infections associated with LA-MRSA. Moreover, there was no published and/or accessible research work in the current study area, Shire, as well as in the region. Thus, the objective of this study was to isolate and identify *S. aureus* from mastitic cows' milk and estimate the occurrence of MRSA.

## 2. Materials and Methods

### 2.1. Study Area

The study was conducted from November 2014 to May 2015 in Shire, Ethiopia. Shire is located at about 1087 km north of Addis Ababa and 304 km from Mekelle at an altitude of 1984 meter above sea level. It is located between 14°6′N 38°17′E/14.100°N 38.283°E latitude and longitude, respectively. The average range of temperature is 25°C–35°C, with 900–1100 mm of annual average range of rainfall. There are four milk shades (dairy farms) containing about 1500 dairy cattle kept in these farms (shades), of which 579 were crossbred dairy cows and the remaining were calves and heifers [18].

### 2.2. Study Design

A cross-sectional type of study was conducted from November 2014 to May 2015 in the Shire town, Northwest of Tigray, Ethiopia, to isolate and identify *S. aureus* from mastitic cows' milk and estimate the occurrence of MRSA strains.

### 2.3. Sample Size and Sample Collection

A total of 220 dairy cows from the four milk shades were selected purposively on the bases of the availability of dairy cows and the willingness and cooperation of the farm owners during sampling. About 10 mL of the milk sample was aseptically collected from each of clinically and subclinically (CMT positive) mastitis nonblind quarters of the selected cows using sterile universal bottles for bacterial isolation according to the study by Quinn et al. [19]. Then, the collected samples were transported in an ice box to the Mekelle University, College of Veterinary Sciences, Microbiology Laboratory for microbiological examination. If immediate inoculation was not convenient, the samples were kept at 4°C for a maximum of 24 h until cultured on standard bacteriological media.

### 2.4. Bacteriological Isolation and Identification

A loop of milk sample was streaked on 5% Sheep Blood Agar (Oxoid, UK), and the plates were incubated aerobically at 37°C and examined after 24 h of incubation for growth. The colonies were provisionally identified on the basis of staining reaction with Gram's stain, cellular morphology, and hemolytic pattern on blood agar. The representative colonies were subcultured on Mannitol Salt Agar (MSA) (Oxoid, UK) and incubated at 37°C. Then, the colonies that grew on MSA were subcultured on nutrient media, and the cultures were preserved and maintained for characterizing the isolates. Catalase test, oxidase test, tube coagulase test, DNase test, and growth on MSA were performed according to the study by Quinn et al. [19], and samples that were considered as positive for coagulase-positive *S. aureus* (CoPS) were further characterized.

### 2.5. Antimicrobial Susceptibility Testing

The *S. aureus* isolates were screened for in vitro antimicrobial susceptibility using the agar disk diffusion method according to the procedure given by Kirby et al. [20] on Mueller–Hinton agar (Oxoid Ltd., Basingstoke, Hampshire, England). It is stated that in the absence of methicillin, the best alternative is to use cefoxitin or oxacillin for MRSA identification [21]. The following eleven different antibiotic discs, with their concentrations given in parentheses, were used in the antibiogram testing: oxacillin (1 *μ*g) (Abtek Biologicals Ltd, Liverpool, UK), vancomycin (30 *μ*g), penicillin G (10 *μ*g), oxytetracycline (30 *μ*g), streptomycin (25 *μ*g), trimethoprim (5 *μ*g), amoxicillin/clavulanic acid (30 *μ*g), ampicillin (10 *μ*g), nalidixic acid (30 *μ*g), compound sulfonamides (300 *μ*g), and kanamycin (30 *μ*g) (Oxoid Company, Hampshire, England). After 24 h of incubation, the clear zones (inhibition zones) of bacterial growth around the antibiotic disc (including the discs) diameter for individual antimicrobial agents were measured and then translated into sensitive (S), intermediate (I), and resistant (R) categories according to the interpretation table of the Clinical and Laboratory Standard Institute [22].

### 2.6. Polymerase Chain Reaction (PCR) for Detection of *mec A* and *fem A* Genes

The *S. aureus* genomic DNA extraction and purification were performed as per the protocol given by Thermo Scientific, GeneJET Genomic DNA Purification Kit for gram-positive organisms. Then, seven of the phenotypically MRSA isolates were screened for the presence of *mec A* and *fem A* genes by multiplex PCR according to the procedure given by Johnson et al. [23] using the following specific primers: F-5′GTA GAA ATG ACT GAA CGT CCG ATA A3′ and R-5′CCA ATT CCA CAT TGT TTC GGT CTA A3′ for *mec* A gene (having a band size of 310 bp) and F-5′-AAA AAA GCA CAT AAC AAG CG-3′ and R-5′GAT AAA GAA GAA ACG AGC AG-3′ for *fem A* gene (*S. aureus* species specific and encoding a factor responsible for methicillin resistance and has an amplicon size of 132 bp). Each PCR reaction mixture (50 *μ*L) was prepared from 5 *μ*L of 10X reaction buffer, 5 *μ*L of template DNA, 1 *μ*L of each primer, 3 *μ*L of 10 mM dNTP mixture, and 1 *μ*L of Taq polymerase. The remaining volume had nuclease-free deionized water. Amplification was carried out in a Tianlong PCR thermocycler with thermal cycling conditions of an initial denaturation at 94°C for 6 min followed by 35 cycles of denaturation at 94°C for 45 s, annealing at 55°C for 30 s and extension at 72°C for 45 s, and with final extension at 72°C for 6 min. Finally, PCR products were separated by running on 1.5% (w/v) agarose gel containing 0.5 g/mL ethidium bromide. Electrophoresis was conducted in a horizontal equipment system for 55 min at 110 V using 1X TAE buffer (40 mM Tris, 1 mM EDTA and 20 mM glacial acetic acid, pH 8.0). The amplicons were visualized under UV-light gel doc, and their molecular weights were estimated by comparing with 100 bp DNA molecular weight marker (Solis BioDyne, Tartu, Estonia) [24].

### 2.7. Data Management and Analysis

All collected data were entered into Microsoft Excel Sheet and analyzed through Statistical Package for Social Sciences, version 16. Accordingly, descriptive statistics such as percentages and frequency distribution were used to determine the prevalence.

## 3. Results

The physical examination of the udder and CMT test results indicated that out of the 220 dairy cows, 64 (29.09%) were positive for bovine mastitis. Bacteriological characterization of these 64 mastitis-positive samples revealed that 21 (32.81%) were found CoPS. The antimicrobial sensitivity test results of the 21 CoPS isolates showed their susceptibility to kanamycin and streptomycin (42.85%). However, 100% of the isolates were resistant to penicillin G, nalidixic acid, and ampicillin, 85.71% to amoxicillin, 47.61% to trimethoprim and oxytetracycline, and 57.14% to compound sulfonamides, and 33.33% of the CoPS were resistant for oxacillin (phenotypically MRSA positive). But 38.09% of the CoPS were found to be resistant and susceptible to vancomycin. Moreover, all the 21 (100%) isolates developed multidrug resistance as it is shown in Table 1.

The PCR amplification of the seven phenotypically MRSA isolates was revealed that 3 (42.9%) of them were found to carry *fem A* gene and 5 (71.4%) of them were found to carry *mec A* genes, which had a molecular weight of 132 bp and 310 bp, respectively.

## 4. Discussion

The overall prevalence of bovine mastitis in the study dairy farms was 29.09%. This finding was more or less in line with the reports of Molalegne et al. [25] in Bahir Dar and its environs, Ethiopia (28.8%), Mulugeta and Wassie [26] in and around Wolaita Sodo, Ethiopia (29.5%), and Bekele et al. [27] in Hawassa Town, Ethiopia (34.3%). However, it was lower than the reports of Indu and Brintya [28] in Silchar Town Dairy Farms, North East India (35.83%), Rgbe et al. [29] in Northern Ethiopia (37.4%), Biniam et al. [30] in and around Wolaita Sodo, Southern Ethiopia (40.9%), Pati and Reena [31] from Northern Plains of India (42.76%), Anueyiagu and Isiyaku [32] in Plateau State, Nigeria (43.75%), Abera et al. [33] in Adama Town, Ethiopia (46.7%), Asmelash et al. [34] in and around Kombolcha, Ethiopia (56%), Tilahun et al. [35] in and around Kombolcha Town, Eastern Amhara, Ethiopia (56%), and Shimels et al. [36] in the Selale/Fitche area, North Showa, Ethiopia (83.1%). The differences in the prevalence of bovine mastitis might be due to the fact that mastitis is a complex disease and associated with different risk factors such as husbandry and management systems of the farms, difference in drug usages and/or treatment, and the geographical locations of the study sites.

The current study revealed 32.81% prevalence of CoPS. This finding was nearly comparable with the findings of Zeryehun et al. [37] in and around Addis Ababa, Ethiopia (28.8%), Joshi et al. [38] in Pokhara, Nepal (29.7%), Suleiman et al. [39] in Plateau State, Nigeria (30.9%), Fitsum [40] in Wolayta Sodo, Ethiopia (32.14%), Muyiwa et al. [41] in Mafikeng town, North-West Province of South Africa (32.5%), Pati and Reena [31] from Northern Plains of India (32.8%), Birhanu et al. [42] in Asella, South Eastern Ethiopia (35.71%), Rgbe et al. [29] in Northern Ethiopia (36%), and Biniam et al. [30] in and around Wolaita Sodo, Southern Ethiopia (37.14%). However, the present finding result was higher than that reported by Riva et al. [43] (9.1%), Gali et al. [44] in Zaria and Kaduna, Nigeria (12.6%), Gali et al. [45] in Nigeria (12.63%), Basanisi et al. [46] in South Italy (12.9%), Abebe et al. [47] around Addis Ababa, Ethiopia (16.2%), Kapllan et al. [48] in Fieri Region in Albania (18%), Fufa et al. [49] in and around Asella, Ethiopia (19.3%), Asiimwe et al. [50] in South-West Uganda (20.3%), Molalegne et al. [25] in Bahir Dar town and its environs, Ethiopia (20.3%), Takele et al. [51] in Addis Ababa, Ethiopia (20.8%), Yodit et al. [52] in Sebeta, Central Oromia, Ethiopia (23.4%), and Mueena et al. [53] in Bangladesh (25.53%); but it was lower than that reported by Sudhanthirakodi et al. [54] in the region of Tirupathi, India (39.09%), Abera et al. [33] in Adama town, Ethiopia (42.14%), Fanta et al. [55] in urban and peri-urban areas of Debre-Zeit, Ethiopia (44%), Ananya and Pranab [56] in Southern Assam, India (47.86%), Deresse et al. [57] in Hawassa area, Ethiopia (48.75%), Jolanta et al. [58] in Poland (50.0%), Shimels et al. [36] in the Selale/Fitche area, North Showa, Ethiopia (51.56%), Gali et al. [44] in Zaria and Kaduna, Nigeria (52.42%), Bekele et al. [27] in Hawassa town, Ethiopia (53.5%), Pant et al. [59] from different regions of Dehradun (60%), Jibril and Huruma [60] in the Morogoro Municipality, Tanzania (64.10%), Purba et al. [61] in Karnal, North India (74.5%), and Indu and Brintya [28] in Silchar Town, North East India (83.72%). These differences in the prevalence of *S. aureus* from the different studies might be due to variations in sample size, isolation techniques, husbandry practices, awareness and skills of the farm owners, animal health delivery systems, and geographic regions of the sampled area.

The antimicrobial sensitivity test results of the current CoPS isolates was almost in agreement with the findings of Asmelash et al. [34] who reported 100% resistant to penicillin G and amoxicillin and 42.7% to cefoxitin; Abera et al. [33] who reported 94.4% resistant to penicillin G and 58.3% to trimethoprim-sulfamethoxazole; Rgbe et al. [29] who reported 82.4% of resistant to ampicillin and 52.9% to trimethoprim-sulfamethoxazole; Abebe et al. [47] who observed 92.2% resistant to penicillin G and 33.3% to oxacillin; Abo-Shama [62] in Sohag Governorate, Egypt, who reported 43.1% resistant to oxacillin and 83.7% to one or more antimicrobial agent; Mueena et al. [53] who reported 100% resistant to penicillin and amoxicillin; Fitsum [40] who reported 93.3% resistant to penicillin G, 53.3% to streptomycin, and 40% to tetracycline; Tilahun et al. [35] who observed 100% resistant to penicillin G and amoxicillin and 42.7% to cefoxitin; Muyiwa et al. [41] who illustrated a large proportion (60%–100%) of resistant to penicillin G, ampicillin, and streptomycin; Ananya and Pranab [56] who reported 87.5% resistant to penicillin G; Pati and Reena [31] who reported 96% resistant to penicillin G and 93% to ampicillin; and Jibril and Huruma [60] who reported 71.74% of resistant to penicillin G and 41.30% to tetracycline.

Moreover, the current result was in line with that of Indu and Brintya [28] who observed 76.78% resistant to penicillin G and 91.07% to nalidixic acid; Biniam et al. [30] who observed 100% resistant to penicillin G, 61.5% to amoxicillin-clavulanic acid, 66.7% to streptomycin, and 71.8% multidrug resistance; Suleiman et al. [39] who reported 35.6% resistant to oxacillin; Fufa et al. [49] who reported 95.5% resistant to penicillin G; Shimels et al. [36] who reported 36.6% MRSA; Sudhanthirakodi et al. [54] who reported 86.04% resistant to penicillin G and 74.42% to ampicillin; Gali et al. [44] who reported 100% of resistant to penicillin G and 46.8% to oxacillin, 55.6% to tetracycline, 44.6% to vancomycin, detected *mec A* by polymerase chain reaction in 4 of the 18 MRSA isolates, and 88.9% multidrug resistance; Gali et al. [45] who reported 100% of resistant to penicillin G, 65% to amoxicillin, and 40% to oxacillin; Yodit et al. [52] who reported 98.5% of resistant to penicillin G; Takele et al. [51] who reported 95.3% of resistant to penicillin G, 88.4% to nalidixic acid, and 100% multidrug resistance; and Fufa et al. [49] who reported 95.5% of resistant to penicillin G and 95.5% multidrug resistance. However, the current finding of MRSA was higher than that reported by Ananya and Pranab [56] (8.93%), Sudhanthirakodi et al. [54] (13.95%), Basanisi et al. [46] (8.3%), Gali et al. [44] (4.8%), and Jibril and Huruma [60] (6.52%). The detection of *mec A* by the PCR is considered a gold-standard technique for oxacillin resistance detection [63]. The resistance of *S. aureus* to penicillin and closely related antibiotics might be attributed to the production of *β*-lactamase, an enzyme that inactivates penicillin and closely related antibiotics. Around 50% of mastitis-causing *S. aureus* strains produce *β*-lactamase [64]. Moreover, the development of antimicrobial resistance might be as a result of repeated therapeutic and/or indiscriminate use of them in the dairy farms, particularly penicillin and oxytetracycline for the treatment of mastitis cases in the study area at large in the country.

## 5. Conclusion and Recommendations

The current study revealed that there is a high prevalence of CoPS as well as MRSA as a cause of bovine mastitis. Though nearly half of the CoPS isolates were susceptible to kanamycin and streptomycin, the high percentage were resistant to penicillin G, nalidixic acid, ampicillin, amoxicillin, trimethoprim, oxytetracycline, compound sulfonamides, oxacillin, and vancomycin. Moreover, all of the CoPS isolates developed multidrug resistance. This higher percentage of multidrug resistance pattern indicates alarming situation for designing prevention and control measures. However, the current study was only targeting *mec A* and *fem A* genes for a few of the phenotypically MRSA-positive isolates for molecular characterization. In general, the detection of CoPS in the milk samples and development of drug resistance indicate that the product is unwholesome for human consumption. Hence, it has a serious economic, animal welfare, food safety, and public health problem. Therefore, strict hygiene should be implemented in the farms by creating awareness among the farm workers, managers, and attendants regarding transmission, zoonotic importance, and control and prevention strategies of the disease; and dispensing of non-prescribed drugs and indiscriminate use of antibiotics should be avoided. Moreover, further studies on molecular characterization and sequencing of MRSA should be conducted by targeting other important genes in addition to the targeted genes in the study area at large in the country.

## Figures and Tables

**Table 1:** In vitro antimicrobial drug susceptibility test results of *S. aureus* isolates.

Antimicrobial discs	Interpretations		
	Susceptible no. (%)	Intermediate no. (%)	Resistant no. (%)
Ampicillin	0	0	21 (100)
Nalidixic acid	0	0	21 (100)
Kanamycin	9 (42.85)	5 (23.8)	7 (33.33)
Trimethoprim	8 (38.09)	3 (14.28)	10 (47.61)
Streptomycin	9 (42.85)	4 (19.04)	8 (38.09)
Oxytetracycline	9 (42.85)	1 (4.76)	10 (47.61)
Amoxicillin	3 (14.285)	0	18 (85.71)
Oxacillin	12 (57.14)	2 (9.52)	7 (33.33)
Penicillin G	0	0	21 (100)
Vancomycin	8 (38.09)	4 (19.04)	8 (38.09)
Compound sulfonamides	1 (4.76)	8 (38.09)	12 (57.14)

## Data Availability

The data used to support the findings of this study are available from the corresponding author upon request.
